# Studies on the Reactivity of (9-Methyl-5,6-dihydronaphtho[1′,2′:4,5]-thieno[2,3-*d*]pyrimidin-11-yl)hydrazine Towards Some Reagents for Biological Evaluation

**DOI:** 10.3797/scipharm.0910-11

**Published:** 2009-12-11

**Authors:** Aymn E. Rashad, Ahmed H. Shamroukh, Randa E. Abdel-Megeid, Hayam H. Sayed, Nayera M. Abdel-Wahed

**Affiliations:** 1 Photochemistry Department, National Research Center, Elbehos Street, Dokki, Cairo, Egypt; 2 Department of Natural and Microbial Products, National Research Center, Elbehos Street, Dokki, Cairo, Egypt

**Keywords:** Thieno[2,3-*d*]pyrimidine, Pyrazole, Pyridazine, *C*-Nucleosides, Antimicrobial activity

## Abstract

(9-Methyl-5,6-dihydronaphtho[1′,2′:4,5]thieno[2,3-*d*]pyrimidin-11-yl)hydrazine (**1**) was used as a precursor for preparation of some novel 1-(9-methyl-5,6-dihydronaphtho[1′,2′:4,5]thieno[2,3-*d*]pyrimidin-11-yl)-1*H*-pyrazoles **2**–**7**, -1*H*-isoindole-1,3(2*H*)-dione **8**, and -pyridazin-3(2*H*)-one **9**. Moreover, the acyclic *C*-nucleosides **10** and **11** were prepared by treating compound **1** with D-glucose. The *in vitro* antimicrobial activity of the tested compounds was evaluated by measuring the zone diameters and some of the prepared products showed potent antimicrobial activity in compared with those of well known drugs (standard). In general, the non-acetylated sugar hydrazone derivative **10** showed the highest antibacterial and antifungal potency among the tested compounds and standard with IZ = 22, 21 and 22 mm and MIC = 62.5 and 31.25 μg/ml, respectively.

## Introduction

Pyrimidine and fused heterocyclic pyrimidine derivatives have attracted a great deal of interest in particular 4-hydrazinopyrimidine derivatives, which were tested for their medicinal, bactericidal and fungicidal activity [1–4]. Pyrimidine and heterocyclic annulated pyrimidine derivatives attracted great interest due to the wide variety of interesting biological activities observed for these compounds, such as anticancer [4], antiviral [5], Anti-HIV-1 Activity [6], anti-inflammatory [7] and antimicrobial activities [8]. In addition, several substituted dihydronaphthothienopyrimidine derivatives showed antimicrobial activities against *Bacillus subtilis*, *Escherichia coli*, *Aspergillus niger* and *Candida albicans* [9], and their ester-containing derivatives demonstrated more antimicrobial activities than the corresponding cyano-containing analogs. In view of the above and in continuation of our research program concerned with structural modification of certain biologically active heterocyclic nuclei with the purpose of enhancing their biological activity [10–16], we aimed to incorporate a fused pyrimidine moiety with other heterocyclic ring system to obtain new functions in an attempt to improve the antimicrobial profile of compounds containing the dihydronaphthothienopyrimidine ring system.

## Results and Discussion

From the view of biological activity, polycondensed fused heteroaromatic systems are often of much greater interest than the constituent monocyclic compounds. The appearance of qualitatively new properties of an annelated molecule, enlargement of the possibility of varying pharmacophore groups in different positions of the molecule and the ability of the latter to interact with various receptors adopting several conformations are apparently of crucial importance [17]. In this investigation, compound **1** [18] was dissolved in ethanol and refluxed with (ethoxymethylene)malononitrile, tetracyanoethylene, [bis(methylthio)methylene]malononitrile, ethyl (ethoxymethylene)cyanoacetate or methyl [bis(methylthio)methylene]cyanoacetate to afford the corresponding substituted pyrazole derivatives **2**–**6**, respectively (Sch. 1). The structures of the latter compounds were confirmed on the basis of their elemental analysis and spectral data (cf. Exp.). The IR spectra of compounds **2**–**4** showed absorption bands characteristic for NH_2_ and C≡N groups, while those of compounds **5** and **6** revealed absorption bands characteristic for NH_2_ and C=O. Also, the ^1^H NMR spectra showed signals at *δ* = 7.10, 6.55, 6.60, 6.81 and 7.00 ppm due to NH_2_ (exchangeable with D_2_O) for compounds **2**–**6**, respectively. The MS gave the molecular ion peaks at *m/z* (%) = 358 (100), 383 (89), 404 (100), 405 (100), and 437 (100) for compounds **2**–**6**, respectively. Similarly, when compound **1** was refluxed with acetyl acetone the pyrazole derivative **7** was obtained (Sch. 1). The ^1^H NMR and ^13^C NMR spectra of the latter compound showed signals at *δ* = 2.05, 2.19 ppm and 11.97, 13.29 ppm for (2CH_3_), while its IR spectrum revealed the absence of NH, NH_2_ groups. The MS, gave the molecular ion peak at *m/z* (%) = 346 (78).

On the other hand, compound **1** was refluxed with tetrachlorophthalic anhydride in glacial acetic acid to afford *N*-imido derivative **8** (Sch. 2). The presence of NH and two C=O groups in the IR spectra and the NH-proton (D_2_O exchangeable) in the ^1^H-NMR spectrum confirmed its structure. Also, the MS of compound **8** gave fragments showing the isotopic pattern due to the presence of chlorine atoms (cf. Exp.).

2-(9-Methyl-5,6-dihydronaphtho[1′,2′:4,5]thieno[2,3-*d*]pyrimidin-11-yl)-6-phenyl-4,5-dihydropyridazin-3(2*H*)-one (**9**) was prepared by heating the hydrazine derivative **1** with β-benzoylpropionic acid. Inspection of the IR spectrum of product **9** showed the presence of the C=O group and its ^1^H NMR spectrum revealed signals at *δ* 2.43, 3.61 ppm for 2CH_2_ of pyridazine ring. In addition, the ^13^C NMR spectrum showed signal at *δ* 173.80 accountable for the C=O group (cf. Exp.).

The hydrazone derivative **10** was prepared by reacting compound **1** with D-glucose in the presence of a catalytic amount of glacial acetic acid. The product **10** revealed absorption bands for OH and NH groups in IR spectra and its ^1^H NMR spectrum showed the presence of the sugar protons, NH, and azo-methine (CH=N) (cf. Exp.).

Acetylation of the hydrazone derivative **10** with acetic anhydride/pyridine at room temperature gave unexpected *O*-acetylated cyclic *C*-nucleosides **11** (Sch. 2). The absence of NH as well as the azo-methine (CH=N) in ^1^H NMR spectra confirmed its structure (cf. Exp.). Also, the ^13^C NMR spectrum showed signals accountable for the acetylated sugar residue (cf. Exp.).

The formation of compound **11** might have taken place *via* oxidative cyclization of the *O*-acetylated sugar. In general, the Dimroth type rearrangement of *S*-triazolopyrimidines was intensively discussed and verified with X-ray diffraction by Rashad et al [16]. So, the triazolo[1,5-*c*]pyrimidine derivative **11** was obtained directly via Dimroth type rearrangement of its triazolo[4,3-*c*]pyrimidine derivative.

## Antimicrobial activity

The *in vitro* antimicrobial activity of the tested compounds was evaluated by measuring the zone diameters and the results were compared with those of well known drugs (standard). Among the tested compounds, for gram-positive and gram-negative bacteria, it was noticed that ß-enaminonitriles of pyrazole ring system **2**–**4** (IZ = 16–18 mm and MIC = 65 μg/ml) demonstrated inhibitory activities more than β-enaminoesters **5** and **6** (IZ = 10–12 mm and MIC = 125 μg/ml). However, the pyridazine derivative **9** and non-acetylated sugar **10** revealed the most significant antibacterial activities (IZ = 20–22 mm and MIC = 62.5 μg/ml). On the other hand, the non-acetylated sugar **10** and the acetylated cyclic *C*-nucleoside **11** revealed more effective antifungal activity than the other tested compounds showing IZ = 22 mm and MIC = 31.25 μg/ml. However, replacement of the hydroxyl moiety in **10** by acetyl group in **11** led to decrease the antibacterial potency of **10** showing IZ = 11, 12 mm, MIC = 62.5 μg/ml and the antifungal activity was not affected. In general, the non-acetylated sugar hydrazone derivative **10** showed the highest antibacterial and antifungal potency among the tested compounds and standard with IZ = 22, 21 and 22 mm and MIC = 62.5 and 31.25 μg/ml, respectively.

## Experimental

All melting points were uncorrected and measured using an Electro-thermal IA 9100 apparatus (Shimadzu, Japan). Microanalytical data were performed by Vario El-Mentar apparatus (Shimadzu, Japan), National Research Centre, Cairo, Egypt. The IR spectra (KBr) were recorded on a Perkin-Elmer 1650 spectrophotometer, National Research Centre, Cairo, Egypt. ^1^H NMR and ^13^C NMR spectra were determined on a Varian Mercury (300 MHz) spectrometer (Varian, UK) and the chemical shifts were expressed in ppm relative to TMS as internal reference, Faculty of science, Cairo University, Egypt. Mass spectra were recorded on 70 eV EI Ms-QP 1000 EX (Shimadzu, Japan), National Research Centre, Cairo, Egypt.

Compound **1** was prepared according to a reported method [18].

### Preparation of compounds 2–6

#### General procedure

To a solution of compound **1** (2.82g, 1mmol) in (20 ml) dry ethanol, (ethoxymethylene)malononitrile, tetracyanoethylene, [bis(methylthio)methylene]malononitrile, ethyl (ethoxymethylene)cyanoacetate or methyl [bis(methylthio)methylene]cyanoacetate (1 mmol) was added and the reaction mixtures were refluxed for 2, 5, 4, 3 and 4 h, respectively. The products, which separated on cooling, were collected by filtration and recrystallized from ethanol to give compounds **2**–**6**.

#### 5-Amino-1-(9-methyl-5,6-dihydronaphtho[1′,2′:4,5]thieno[2,3-*d*]pyrimidin-11-yl)-1*H*-pyrazole-4-carbonitrile (**2**)

Yield (95%), M.p. 260–262°C; IR (KBr) *ν* = 3407, 3200 (NH_2_), 2209 (CN) cm^−1; 1^H NMR (DMSO-d_6_) *δ* = 2.77 (s, 3H, CH_3_), 2.95–3.00 (m, 4H, 2CH_2_), 6.34 (d, *J* = 8 Hz, 1H, Ar-H), 6.91–7.31 (m, 5H, 3Ar-H and NH_2_, D_2_O exchangeable), 7.55 (s, 1H, C_3_-H); ^13^C NMR (DMSO-d_6_) *δ* = 25.32 (CH_3_), 25.67 (C-5′), 29.62 (C-6′), 115.29 (C-4), 118.72 (CN), 125.21, 126.83, 127.29, 128.12, 128.47 (Ar-C), 131.89 (C-3), 135.48 (C-6a′), 141.92 (C-11c′), 143.35 (C-11b′), 150.45 (C-7a′), 153.64 (C-5), 162.16 (C-9′), 172.10 (C-11′); MS, *m/z* (%): 358 (M^+^, 100). Anal. calcd for C_19_H_14_N_6_S: C, 63.67; H, 3.94; N, 23.45; S, 8.95. Found: C, 63.62; H, 4.00; N, 23.39; S, 9.01.

#### 5-Amino-1-(9-methyl-5,6-dihydronaphtho[1′,2′:4,5]thieno[2,3-*d*]pyrimidin-11-yl)-1*H*-pyrazole-3,4-dicarbonitrile (**3**)

Yield (65%), M.p. 166–168 °C; IR (KBr) *ν* = 3407, 3200 (NH_2_), 2219 (CN) cm^−1; 1^H NMR (DMSO-d_6_) *δ* = 2.80 (s, 3H, CH_3_), 3.00–3.15 (m, 4H, 2CH_2_), 6.25 (d, *J* = 8 Hz, 1H, Ar-H), 6.55 (s, 2H, NH_2_, D_2_O exchangeable), 7.00–7.26 (m, 3H, Ar-H); ^13^C NMR (DMSO-d_6_) *δ* = 25.14 (CH_3_), 25.23 (C-5′), 29.18 (C-6′), 110.68 (CN), 116.69 (CN), 124.34, 125.90, 127.25, 128.05 (Ar-C), 131.05 (C-3), 134.59 (C-6a′), 142.71 (C-11c′), 148.35 (C-11b′), 150.40 (C-7a′), 152.69 (C-5), 162.16 (C-9′), 173.10 (C-11′); MS, *m/z* (%): 383 (M^+^, 89). Anal. calcd for C_20_H_13_N_7_S: C, 62.65; H, 3.42; N, 25.57; S, 8.36. Found: C, 62.60; H, 3.48; N, 25.54; S, 8.40.

#### 5-Amino-1-(9-methyl-5,6-dihydronaphtho[1′,2′:4,5]thieno[2,3-*d*]pyrimidin-11-yl)-3-(methylsulfanyl)-1*H*-pyrazole-4-carbonitrile (**4**)

Yield (90%), M.p. 124–126 °C; IR (KBr) *ν* = 3460, 3346 (NH_2_), 2215 (CN) cm^−1; 1^H NMR (DMSO-d_6_) *δ* = 1.58 (s, 3H, SCH_3_), 2.80 (s, 3H, CH_3_), 2.90–3.05 (m, 4H, 2CH_2_), 6.50 (d, *J* = 8 Hz, 1H, Ar-H), 6.60 (s, 2H, NH_2_, D_2_O exchangeable), 7.00–7.25 (m, 3H, Ar-H); ^13^C NMR (DMSO-d_6_) *δ* = 11.64 (CH_3_), 25.15 (CH_3_), 25.34 (C-5′), 29.32 (C-6′), 113.04 (C-4), 115.59 (CN), 126.03, 126.16, 126.47, 127.13, 128.51 (Ar-C), 132.50 (C-3), 133.82 (C-6a′), 140.67 (C-11c′), 150.75 (C-11b′), 151.25 (C-7a′), 153.83 (C-5), 160.17 (C-9′), 172.31 (C-11′); MS, *m/z* (%): 404 (M^+^, 100). Anal. calcd for C_20_H_16_N_6_S_2_: C, 59.38; H, 3.99; N, 20.78; S, 15.85. Found: C, 59.29; H, 4.05; N, 20.71; S, 15.92.

#### Methyl 5-amino-1-(9-methyl-5,6-dihydronaphtho[1′,2′:4,5]thieno[2,3-*d*]pyrimidin-11-yl)-1*H*-pyrazole-4-carboxylate (**5**)

Yield (90%), M.p. 139–141 °C; IR (KBr) *ν* = 3464, 3354 (NH_2_), 1685 (C=O) cm^−1; 1^H NMR (DMSO-d_6_) *δ* = 1.34 (t, *J =* 6.9 Hz, 3H, CH_3_), 2.81 (s, 3H, CH_3_), 2.96–3.07 (m, 4H, 2CH_2_), 4.28 (q, *J* = 7.5 Hz, 2H, CH_2_), 6.43 (d, *J* = 8 Hz, 1H, Ar-H), 6.81 (s, 2H, NH_2_, D_2_O exchangeable), 6.90–7.35 (m, 4H, 3Ar-H and C_3_-H); ^13^C NMR (DMSO-d_6_) δ 14.45 (CH_3_), 25.17 (CH_3_), 25.29 (C-5′), 29.32 (C-6′), 59.82 (O*C*H_2_), 96.49 (C-4), 125.65, 126.03, 126.25, 127.33, 128.30 (Ar-C), 131.85 (C-3), 134.14 (C-6a′), 140.77 (C-11c′), 141.41 (C-11b′), 151.35 (C-7a′), 151.83 (C-5), 160.39 (C-9′), 164.31 (C-11′), 172.29 (C=O); MS, *m/z* (%): 405 (M^+^, 100). Anal. calcd for C_21_H_19_N_5_O_2_S: C, 62.20; H, 4.72; N, 17.27; S, 7.91. Found: C, 62.30; H, 4.65; N, 17.33; S, 7.81.

#### Ethyl 5-amino-1-(9-methyl-5,6-dihydronaphtho[1′,2′:4,5]thieno[2,3-*d*]pyrimidin-11-yl)-3-(methylsulfanyl)-1*H*-pyrazole-4-carboxylate (**6**)

Yield (90%), M.p. 250–252 °C; IR (KBr) *ν* = 3460, 3346 (NH_2_), 1685 (C=O) cm^−1; 1^H NMR (DMSO-d_6_) *δ* = 1.45 (s, 3H, SCH_3_), 2.77 (s, 3H, CH_3_), 2.97–3.10 (m, 4H, 2CH_2_), 3.83 (s, 3H, OCH_3_), 6.51 (d, *J* = 8 Hz, 1H, Ar-H), 6.97–7.23 (m, 5H, 3Ar-H and NH_2_, D_2_O exchangeable); ^13^C NMR (DMSO-d_6_) *δ* = 11.16 (SCH_3_), 25.12 (CH_3_), 25.34 (C-5′), 29.37 (C-6′), 51.03 (OCH_3_), 93.95 (C-4), 125.92, 126.27, 126.53, 126.85, 128.78 (Ar-C), 132.76 (C-3), 133.59 (C-6a′), 139.92 (C-11c′), 150.86 (C-11b′), 151.46 (C-7a′), 153.56 (C-5), 160.06 (C-9′), 164.52 (C-11′), 172.04 (C=O); MS, *m/z* (%): 437 (M^+^, 100). Anal. calcd for C_21_H_19_N_5_O_2_S_2_: C, 57.65; H, 4.38; N, 16.01; S, 14.66. Found: C, 57.71; H, 4.33; N, 16.09; S, 14.60.

#### 11-(3,5-Dimethyl-1H-pyrazol-1-yl)-9-methyl-5,6-dihydronaphtho[1′,2′:4,5]-thieno[2,3-d]pyrimidine (7)

To a solution of compound **1** (2.82 g, 1 mmol) in ethanol (20 ml), acetyl acetone (1 mmol) was added and the reaction mixture was refluxed for 10 h. The solvent was then removed under reduced pressure and the residue was recrystallized from ethanol to give compound **7**. Yield (70%), M.p. 129–131 °C; ^1^H NMR (DMSO-d_6_) *δ* = 2.05 (s, 3H, CH_3_), 2.19 (s, 3H, CH_3_), 2.86 (s, 3H, CH_3_), 2.95–3.03 (m, 4H, 2CH_2_), 5.91 (s, 1H, C_4′_-H), 6.10 (d, *J* = 8 Hz, 1H, Ar-H), 6.79–7.19 (m, 3H, Ar-H); ^13^C NMR (DMSO-d_6_) *δ* = 11.97 (CH_3_), 13.29 (CH_3_), 25.04 (CH_3_), 25.36 (C-5), 29.41 (C-6), 108.56 (C-4′), 119.21, 124.89, 126.19, 126.33, 127.15, 127.82 (Ar-C), 131.06 (C-3′), 134.49 (C-6a), 140.49 (C-11c), 141.58 (C-11b), 151.03 (C-7a), 161.85 (C-9), 172.04 (C-11); MS, *m/z* (%): 346 (M^+^, 78). Anal. calcd for C_20_H_18_N_4_S: C, 69.34; H, 5.24; N, 16.17; S, 9.26. Found: C, 69.30; H, 5.29; N, 16.09; S, 9.33.

#### 4,5,6,7-Tetrachloro-2-[(9-methyl-5,6-dihydronaphtho[1′,2′:4,5]thieno[2,3-d]pyrimidin-11-yl)amino]-1H-isoindole-1,3(2H)-dione (8)

A mixture of compound **1** (2.82 g, 1 mmol) and tetrachlorophthalic anhydride (2.85 g, 1 mmol) in glacial acetic acid (50 ml) was refluxed for 2 h. The formed precipitate was filtered on hot, dried and recrystallized from dioxane to give compound **8**. Yield (73%), M.p. 285–287 °C; IR (KBr) *ν* = 3326 (NH) and 1791, 1724 (anhydride CO) cm^−1; 1^H NMR (DMSO-d_6_) *δ* = 2.35 (s, 3H, CH_3_), 2.83–3.28 (m, 4H, 2CH_2_), 7.08–7.35 (m, 2H, Ar-H), 7.85 (d, *J* = 7.2 Hz, 1H, Ar-H), 8.37 (d, *J* = 7.6 Hz, 1H, Ar-H), 9.60 (s, 1H, NH, D_2_O exchangeable); ^13^C NMR (DMSO-d_6_) *δ* = 25.10 (CH_3_), 26.02 (C-5′), 30.13 (C-6′), 98.95 (C-2a), 125.90, 126.27, 126.71, 126.85, 128.78 (Ar-C), 132.35 (C-3), 134.82 (C-6a′), 139.92 (C-11c′), 150.68 (C-11b′), 154.46 (C-7a′), 155.50 (C-6a), 160.06 (C-9′), 162.52 (C-11′), 164.10 (C=O), 168.50 (C=O); MS, *m/z* (%): 552 (M^+^, Cl^37^, 11.32), 550 (M^+^, Cl^35^, 4.72). Anal. calcd for C_23_H_12_Cl_4_N_4_O_2_S: C, 50.20; H, 2.20; Cl, 25.77; N, 5.82; S, 5.83. Found: C, 50.31; H, 2.12; Cl, 25.81; N, 5.78; S, 5.90.

#### 2-(9-Methyl-5,6-dihydronaphtho[1′,2′:4,5]thieno[2,3-d]pyrimidin-11-yl)-6-phenyl-4,5-dihydropyridazin-3(2H)-one (9)

A mixture of compound **1** (2.82 g, 1 mmol) and β-benzoylpropionic acid (1.78 g, 1 mmol) in ethanol (50 ml) was refluxed for 4 h. The formed precipitate was filtered off hot, dried and recrystallized from dioxane to give compound **9**. Yield (97%), M.p. 154–156 °C; IR (KBr) *ν* = 1691 (C=O) cm^−1; 1^H NMR (DMSO-d_6_) *δ* = 2.43 (m, 5H, CH_2_ and CH_3_), 2.81–2.95 (m, 4H, 2CH_2_), 3.61 (t, *J* = 7.5 Hz, 2H, CH_2_), 7.10–7.45 (m, 9H, Ar-H); ^13^C NMR (DMSO-d_6_) *δ* = 17.74 (CH_3_), 23.87 (C-5′), 29.35 (C-6′), 39.92 (C-5), 56.64 (C-4), 125.30, 125.85, 126.53, 127.62, 128.45 (Ar-C), 131.24 (C-3), 134.66 (C-6a′), 139.58 (C-11c′), 150.21 (C-11b′), 154.40 (C-7a′), 160.25 (C-9′), 162.51 (C-11′), 173.80 (C=O); MS, *m/z* (%): 424 (M^+^, 100). Anal. calcd for C_25_H_20_N_4_OS: C, 70.73; H, 4.75; N, 13.20; S, 7.55. Found: C, 70.66; H, 4.81; N, 13.12; S, 7.62.

#### D-Glucose (9-methyl-5,6-dihydronaphtho[1′,2′:4,5]thieno[2,3-d]pyrimidin-11-yl)hydrazone (10)

A mixture of compound **1** (2.82 g, 1 mmol), D-glucose (1.80 g, 1 mmol), ethanol (30 ml), and a catalytic amount of glacial acetic acid (3 drops) was heated at 80 °C for 2 h. The formed precipitate was filtered off, dried and recrystallized from ethanol to give compound **10**. Yield (60%), M.p. 142–144 °C; IR (KBr) *ν* = 3353–3220 (broad, OH+NH) cm^−1; 1^H NMR (DMSO-d_6_) *δ* = 2.40 (m, 3H, CH_3_), 2.80–3.02 (m, 4H, 2CH_2_), 3.20–3.60 (protons of the alditol congregated with the solvent absorption) [14], 3.70–3.80 (m, 2H, *CH_2_*OH), 4.40-5.11 (m, 5H, 5OH, D_2_O exchangeable), 7.05–7.42 (m, 5H, Ar-H and NH, D_2_O exchangeable), 8.30 (s, 1H, N=CH); ^13^C-NMR (DMSO-d_6_) *δ* = 18.82 (CH_3_), 25.60 (C-5), 29.63 (C-6), 61.26, 70.71, 72.78, 77.12, 92.65 (C-alditol), 125.91, 126.20, 126.75, 126.80, 128.74 (Ar-C), 134.62 (C-6a′), 139.91 (C-11c′), 150.52 (C-11b′), 153.42 (C-7a′), 160.06 (C-9′), 162.51 (C-11′), 162.90 (N=*C*H). Anal. calcd for C_21_H_24_N_4_O_5_S: C, 56.74; H, 5.44; N, 12.60; S, 7.21. Found: C, 56.84; H, 5.39; N, 12.69; S, 7.11.

#### (1S)-1,2,3,4,5-Penta-O-acetyl-1-C-(5-methyl-8,9-dihydronaphtho[1′,2′:4,5]-thieno[3,2-e][1,2,4]triazolo[1,5-c]pyrimidin-2-yl)-D-arabinitol (11)

A solution of compounds **10** (1 mmol) in a mixture of acetic anhydride (10 mL) and anhydrous pyridine (10 ml) was stirred at room temperature for 24 h. The reaction mixture was poured into ice-water with stirring and the solids that precipitated were collected by filtration, washed with water, dried and recrystallized from ethanol to give compounds **11**. Yield (70%), M.p. 109–111 °C; IR (KBr) *ν* = 1748 (OAc), 1602 (C=N); ^1^H NMR (DMSO-d_6_) *δ* = 1.80–2.10 (m, 15H, 5OAc), 2.40 (m, 3H, CH_3_), 2.80–3.10 (m, 6H, 3CH_2_), 4.20–5.50 (m, 4H, 4C*H*OAc), 7.10–7.40 (m, 4H, Ar-H); ^13^C-NMR (DMSO-d_6_) *δ* = 20.50, 20.62, 20.66, 20.74, 20.88 (5CH_3_), 22.70 (C-5′), 29.86 (C-6′), 61.41, 65.90, 67.08, 67.52, 70.52, 73.99 (C-alditol), 109.91 (C-4), 126.81, 127.24, 127.38, 127.53 (Ar-C), 135.13 (C-3), 136.62 (C-6a′), 142.12 (C-11c′), 149.52 (C-11b′), 153.40 (C-7a′), 160.10 (C-9′), 162.51 (C-11′), 169.56, 170.18, 170.59, 170.62, 170.83 (5CO). Anal. calcd for C_32_H_34_N_4_O_10_S: C, 57.65; H, 5.14; N, 8.40; S, 4.81. Found: C, 57.55; H, 5.20; N, 8.45; S, 4.80.

### Antimicrobial activity

#### In vitro antimicrobial screening

The newly synthesized compounds were screened for their antibacterial activity against one gram positive bacteria*, Bacillus subtilis* NRRL B-543 and one gram negative bacteria*, Escherichia coli* NRRL B-210 and yeast *Candida albican*s NRRL Y-477. These microorganisms were obtained from Northern Utilisation Research and Development Division, U.S. Departement of Agricultural Peoria, Illinois, USA. Chloramphenicol and Fluconazole were purchased (pure form) from Egyptian market and used in a concentration of 2 mg/ml as references for antibacterial and antifungal activities. These compounds were assayed by the agar diffusion method [19]. The assay medium flasks containing 50 ml of nutrient agar medium for bacteria and Czapek’s-Dox agar media for yeast. The holes each of 9 mm diameter were made by scooping out medium with a sterilized cork borer in a petri dish which was seeded with the organisms. The solutions of each test compound (0.10 ml) were added separately in the holes and Petri dishes were subsequently incubated. The incubation was carried out at 30°C for 24h. Simultaneously, controls were maintained by employing 0.10 ml of dimethylsulfoxide (DMSO) which did not reveal any inhibition and zones of inhibition produced by each compound was measured in mm. The results of antimicrobial studies are given in Table 1.

#### Minimal inhibitory concentration (MIC) measurement

The bacteriostatic activity of the active compounds (having inhibition zones (IZ) ≥ 16 mm) was then evaluated using the two fold serial dilution technique [20]. Two fold serial dilutions of the test compounds and reference drugs solutions were prepared using the proper nutrient broth. The final concentration of the solutions varied between 500 and 7.81 μg/ml with the concentration of DMF not exceeding 2.5%. Each 0.10 ml from the tested compounds in DMF was mixed with 1 ml, 2 ml, and 3 ml sterilized distilled water and 0.10 ml from each diluted samples was added to test tubes. The tubes were then inoculated with the test organisms, grown in their suitable broth at 37 °C for 24 hours for bacteria and 48 hours for fungi (about 1×10^6^ cells/ml), each 5 ml received 0.10 ml of the above inoculum and were incubated at 37 °C for 48 hours. The lowest concentration showing no growth was taken as the minimum inhibitory concentration (MIC) (Table 2).

## Conclusions

The overall results indicated that, the tested compounds showed promising antimicrobial activity against bacteria and Fungi. Among the tested compounds, for gram-positive and gram-negative bacteria, it was noticed that ß-enaminonitriles of pyrazole ring system **2**, **3** and **4** demonstrated inhibitory activities more than ß-enaminoesters **5** and **6**. However, the pyridazine derivative **9** and non-acetylated sugar **10** revealed the most significant antibacterial activities. On the other hand, the non-acetylated sugar **9** and the acetylated cyclic *C*-nucleoside **10** revealed more effective activity against yeast than the other tested compounds.

## Supporting Information

The scanned ^1^H NMR spectra of compounds **2** and **4**, and the scanned ^13^C NMR spectra of compounds **2**, **4** and **7** are available in the online version (Format: PDF, Seize: ca. 0.3 MB): http://dx.doi.org/10.3797/scipharm.0910-11.

## Figures and Tables

**Sch. 1.**
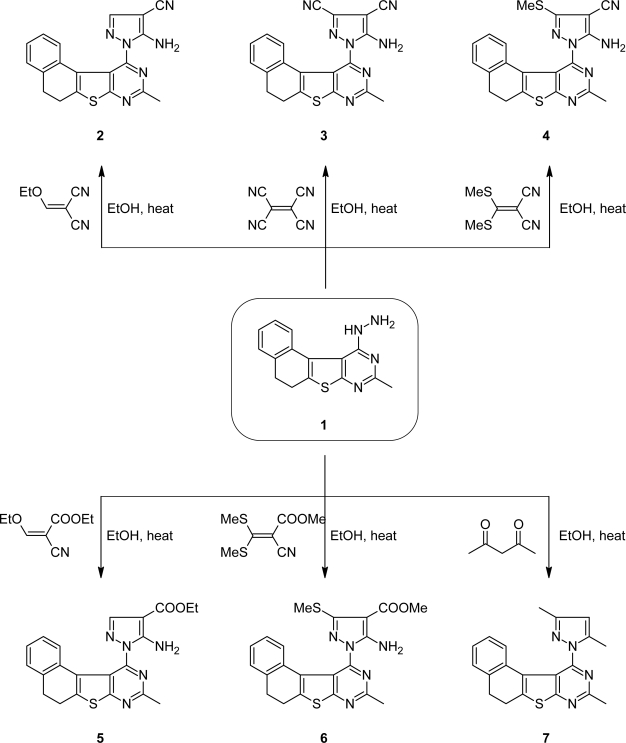


**Sch. 2.**
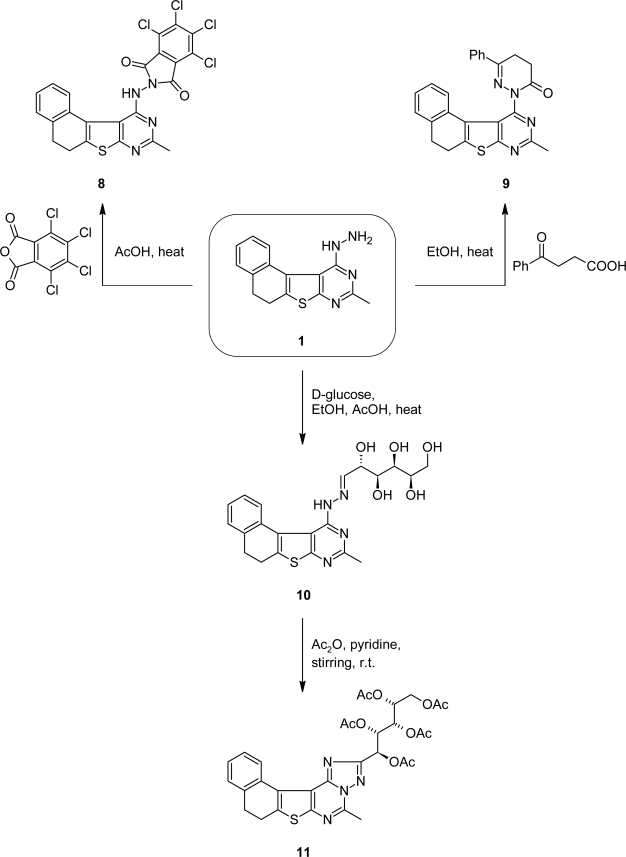


**Tab. 1.** The inhibition zones diameter (IZ) in mm.

**Compound No.**	***Escherichia coli***	***Bacillus subtilis***	***Candida albicans***
**2**	16	16	11
**3**	16.5	16	12
**4**	16	18	16
**5**	12	11	11
**6**	11	10	10
**7**	11	12	10
**8**	12	12	10
**9**	20	20	15
**10**	22	21	22
**11**	12	11	22
Chloramphenicol	24	24	–
fluconazole	–	–	26

Highly active (inhibition zone > 20 mm); Moderately active (inhibition zone16–19 mm); Slightly active (inhibition zone 11–15 mm); (–) no inhibition zone

**Tab. 2.** MIC in μg/ml of the most active compounds.

**Compound No.**	***Escherichia coli***	***Candida albicans***	***Bacillus subtilis***

4	65	31.25	65
5	125	31.25	125
9	62.5	15	62.5
10	62.5	31.25	62.5
11	12	31.25	11
Chloramphenicol	10	–	10
Fluconazole	–	0.5	–
