# Supplementary material for: Studies on the Reactivity of (9-Methyl-5,6-dihydronaphtho[1′,2′:4,5]-thieno[2,3-d]pyrimidin-11-yl)hydrazine Towards Some Reagents for Biological Evaluation
Source: Sci Pharm. 2009 Dec 11;78(1):1–12. doi: 10.3797/scipharm.0910-11 (PMC3002830; doi:10.3797/scipharm.0910-11)
Supplement: Supplementary file 1 — The scanned 1H NMR spectra of compounds 2 and 4, and the scanned 13C NMR spectra of compounds 2, 4 and 7 are available in the online version (Format: PDF, Seize: ca. 0.3 MB): http://dx.doi.org/10.3797/scipharm.0910-11. [file scipharm.2010.78.1_supp_info.pdf]

Scientia Pharmaceutica www.scipharm.at

Open Access

Supporting Information to

Studies on the Reactivity of (9-Methyl-5,6-dihydronaphtho[1',2':4,5]-thieno[2,3-*d*]pyrimidin-11-yl)hydrazine Towards Some Reagents for Biological Evaluation

Aymn E. Rashad, Ahmed H. Shamroukh, Randa E. Abel-Megeid, Hayam H. Sayed, Nyera M. Abel-Wahed

Published in Sci Pharm. 2010; 78: 1–12 doi:10.3797/scipharm.0910-11

Available from: http://dx.doi.org/10.3797/scipharm.0910-11

© Rashad *et al.*; licensee Österreichische Apotheker-Verlagsgesellschaft m. b. H., Vienna, Austria.

This is an Open Access article distributed under the terms of the Creative Commons Attribution License (http://creativecommons.org/licenses/by/3.0/), which permits unrestricted use, distribution, and reproduction in any medium, provided the original work is properly cited.

Table of Contents

Fig. S1. ^{1}H NMR spectrum of compound **2**.
Fig. S2. ^{13}C NMR spectrum of compound **2**.
Fig. S3 ^{1}H NMR spectrum of compound **4**.
Fig. S4 ^{13}C NMR spectrum of compound **4**.
Fig. S5. ^{13}C NMR spectrum of compound **7**.

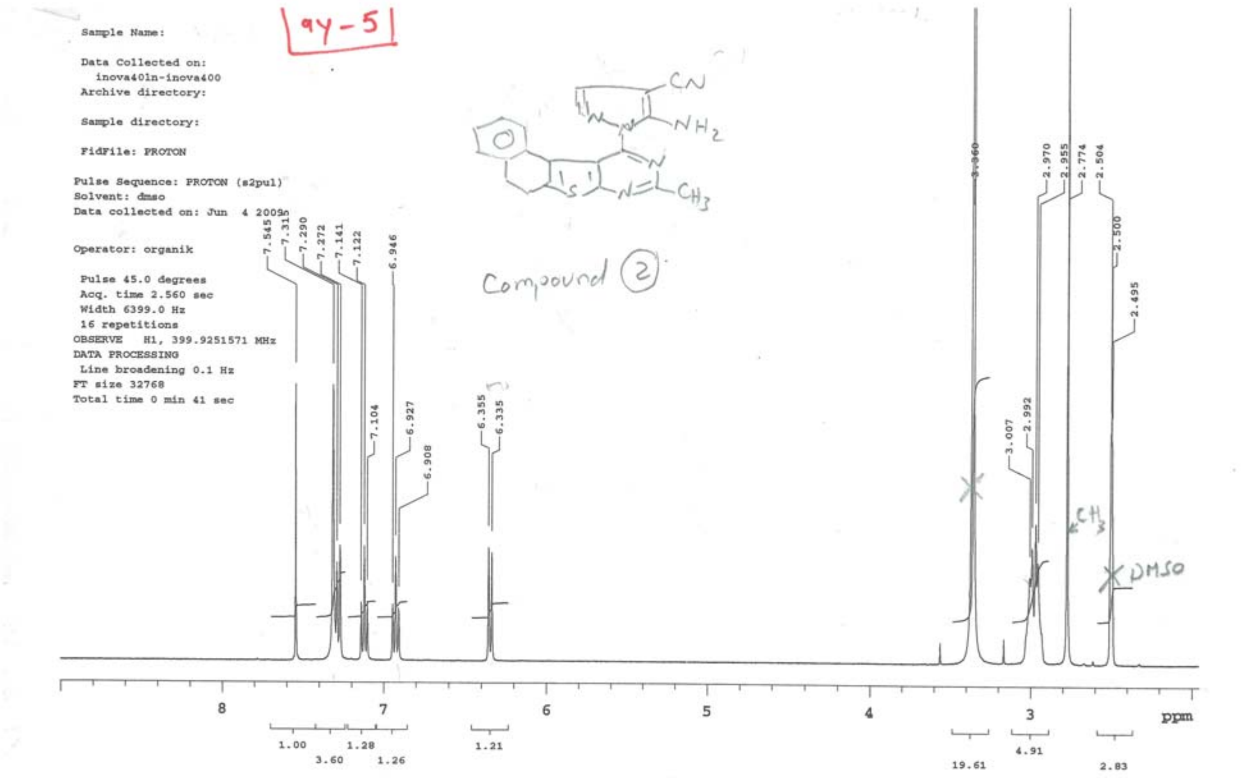

Fig. S1. ^{1}H NMR spectrum of compound **2**.

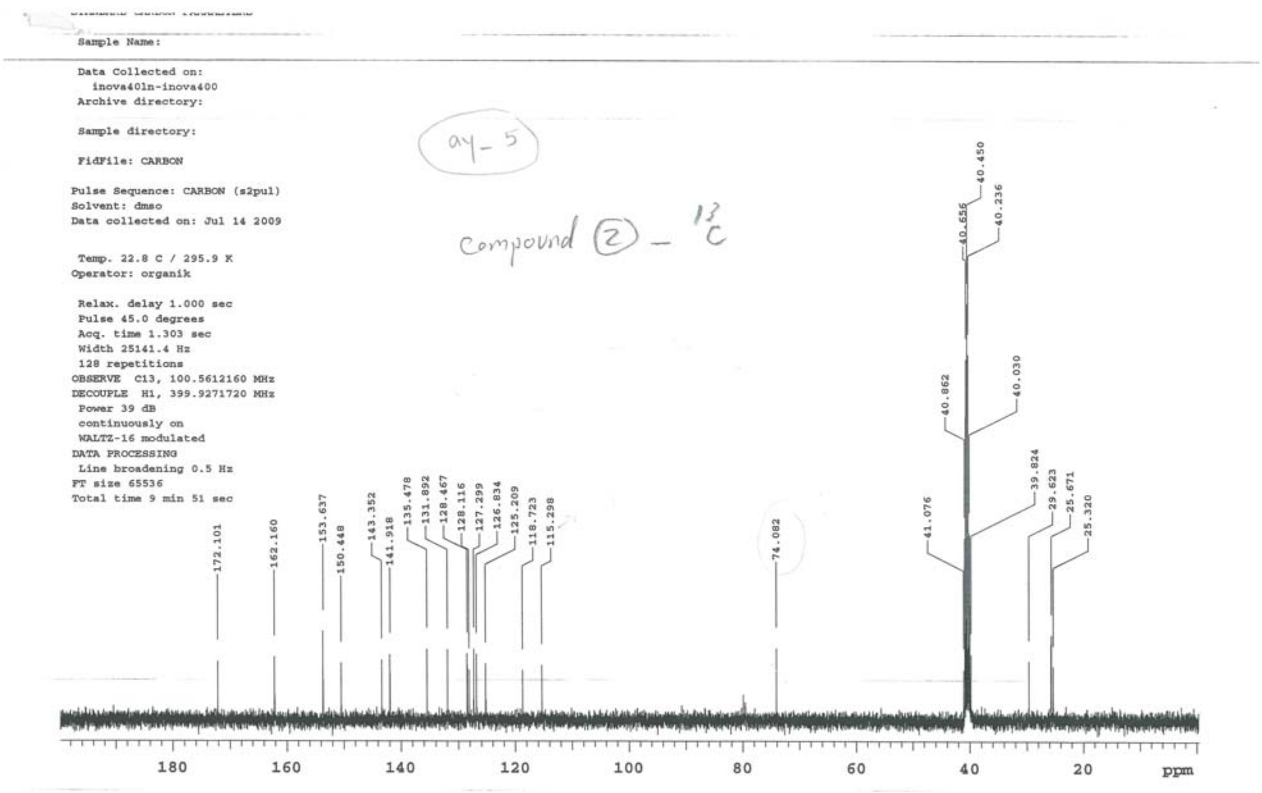

Fig. S2. ^{13}C NMR spectrum of compound **2**.

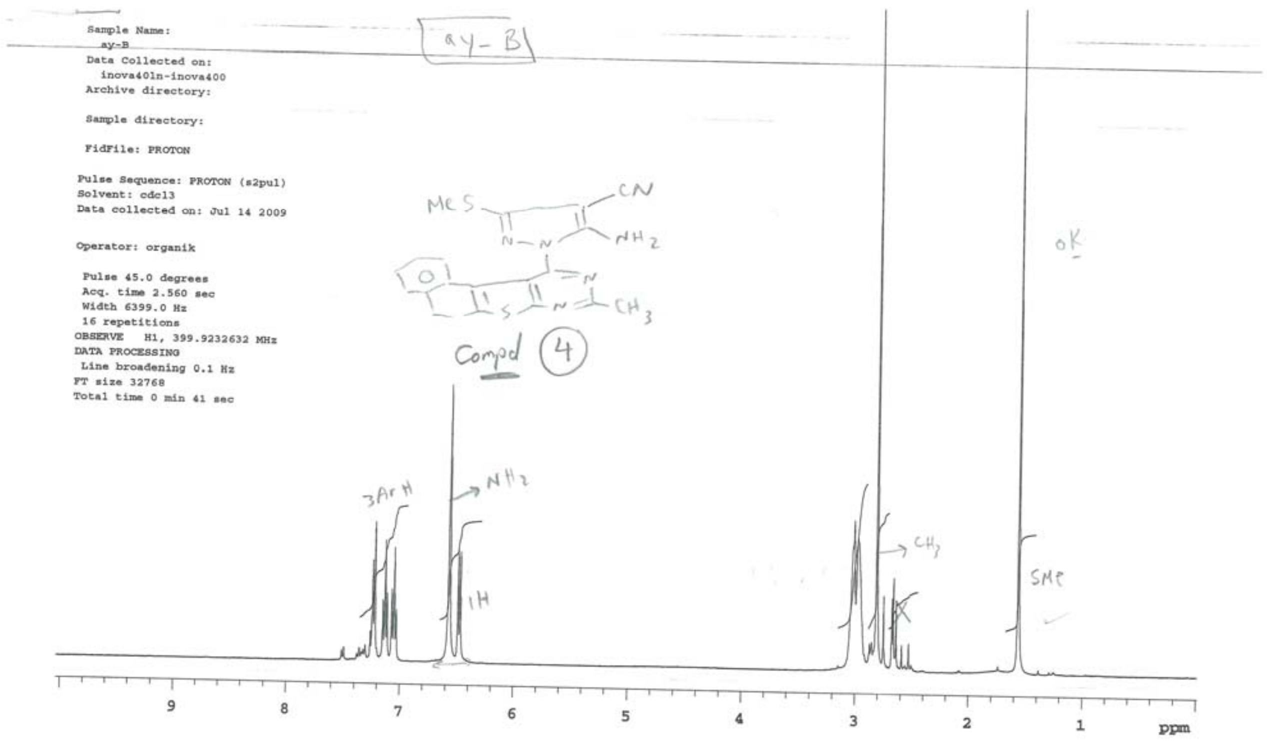

Fig. S3. ^{1}H NMR spectrum of compound **4**.

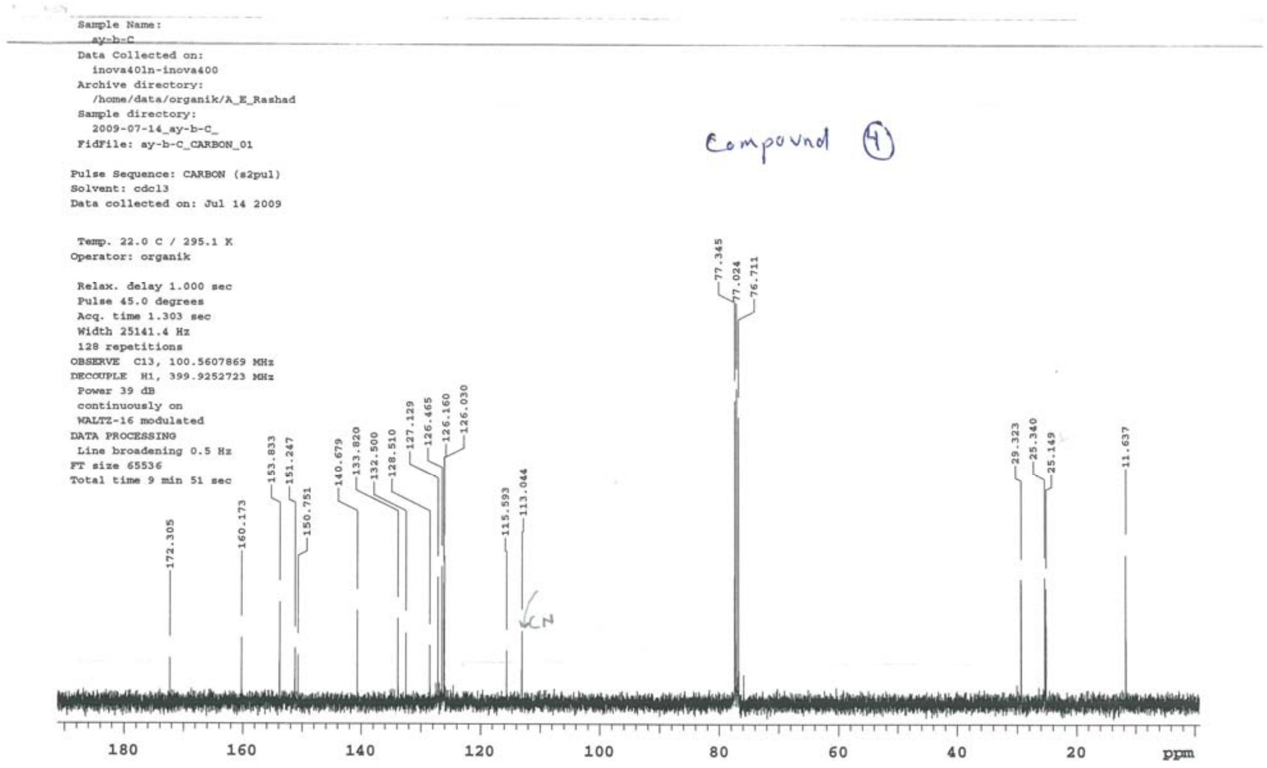

Fig. S4. ^{13}C NMR spectrum of compound 4.

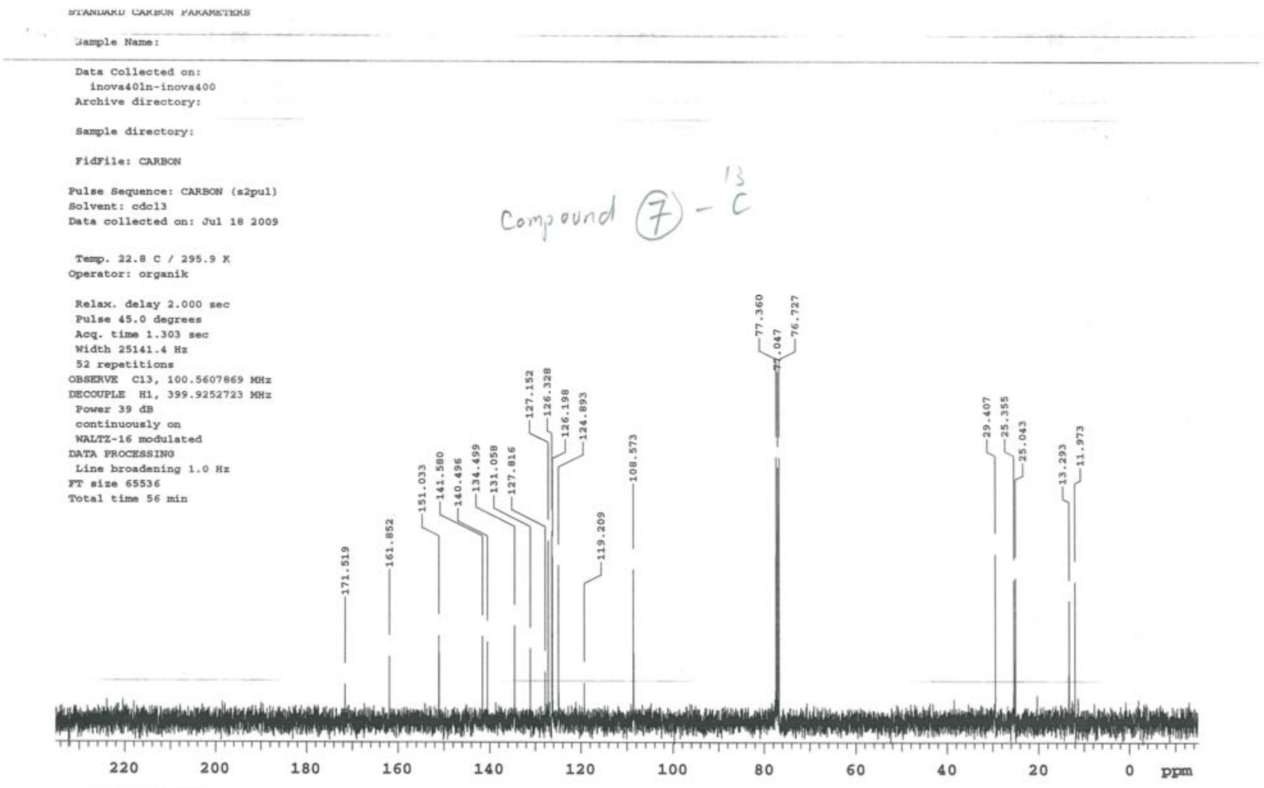

Fig. S6. ^{13}C NMR spectrum of compound **7**.
